# Nucleic Acid-Rich Stress Granules Are Not Merely Crowded Condensates: A Quantitative Raman Imaging Study

**DOI:** 10.1021/acs.analchem.4c01096

**Published:** 2024-10-15

**Authors:** Ren Shibuya, Shinji Kajimoto, Hideyuki Yaginuma, Tetsuro Ariyoshi, Yasushi Okada, Takakazu Nakabayashi

**Affiliations:** § Graduate School of Pharmaceutical Sciences, Tohoku University, Aoba-ku, Sendai 980-8578, Japan; ‡ JST PRESTO, Kawaguchi, Saitama 332-0012, Japan; † Department of Cell Biology and Physics, Universal Biology Institute and International Research Center for Neurointelligence, The University of Tokyo, Bunkyo-ku, Tokyo 113-0033, Japan; # Laboratory for Cell Polarity Regulation, RIKEN Center for Biosystems Dynamics Research, Suita, Osaka 565-0874, Japan

## Abstract

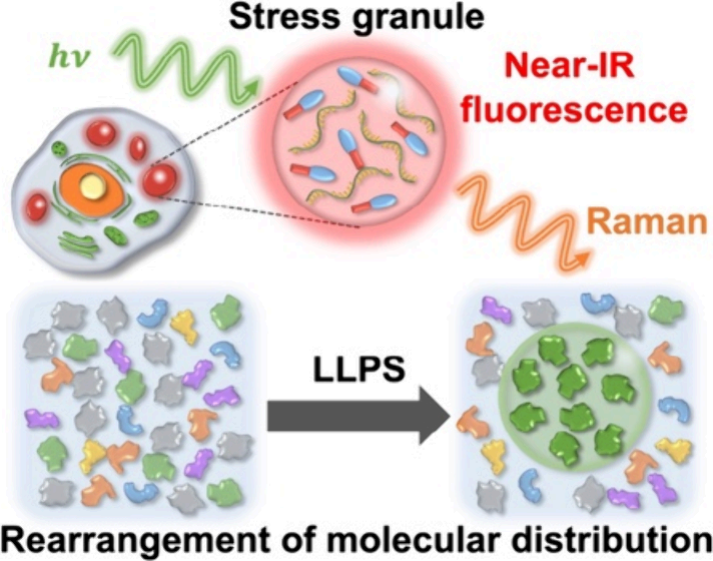

Liquid droplets, formed by intracellular liquid–liquid phase separation (LLPS), are called membraneless organelles. They provide transient enzymatic reaction fields for maintaining cellular homeostasis, although they might transform into aggregates, leading to neurodegenerative diseases. To understand the nature of intracellular droplets, it is crucial to quantify the liquid droplets inside a living cell as well as to elucidate the underlying biological mechanism. In this study, we performed near-infrared fluorescence and Raman imaging to quantify chemical components inside stress granules (SGs) formed by LLPS in living cells. The Raman images reveal that the nucleic acid concentration inside the SGs was more than 20% higher than the surrounding cytoplasm, whereas the lipid concentration was lower. Quantitative Raman intensity analysis using a water Raman band as an internal standard enables *in situ* concentration determination of nucleic acids in the SGs and other organelles. The intensity of the biomolecular C–H bands relative to the water band indicates that the crowding environment inside the SGs depends on the stress type; under oxidative stress, the inside of the SGs was nearly identical to the outside, whereas it was sparser in hyperosmotic stressed cells, suggesting that the high concentrations of nucleic acids play a pivotal role in maintaining the environments inside the SGs. These results demonstrate that intracellular droplets are not always highly condensed.

## Introduction

Liquid–liquid phase separation (LLPS), where a multicomponent solution separates into dense and sparse liquid phases, plays a crucial role in cell compartmentalization.^1−4^ The intracellular liquid droplets formed by LLPS are sometimes called membraneless organelles, provide a unique and transient reaction field for intracellular events.^5,6^ However, liquid droplets can be the precursors to the aggregation and fibril formation of proteins, leading to the pathogenesis of neurodegenerative diseases.^7,8^ Understanding the properties of liquid droplets formed by LLPS is crucial for revealing various physiological events in cells and the pathogenesis of neurodegenerative diseases.

Quantitative analysis of the chemical components and their concentrations in a liquid droplet is important for understanding the properties and functions of the droplet. Studies on LLPS have been conducted by labeling a target biomolecule with a fluorescent tag and observing the fluorescent droplets formed by the labeled molecule. Fluorescence imaging, however, reveals only the presence of labeled molecules, not whether unlabeled molecules are present in the droplets. Additionally, photobleaching of fluorescent tags complicates the concentration quantification of the target molecule. Intracellular droplets are one of the molecular crowding environments, representing crowded states with biomolecules.^9−11^ However, it is challenging to assess the differences in the crowding environments inside and outside the droplets using fluorescence; there is no direct method to quantify the environments inside liquid droplets and the surrounding regions.

Raman imaging is a promising method for visualizing the chemical components inside a living cell in a label-free and noninvasive manner.^12,13^ Raman imaging using different Raman bands offers simultaneous visualization of the distributions of various molecules. Furthermore, as shown in our previous reports, the concentration of biomolecules can be evaluated quantitatively using a strong water Raman band as an internal standard.^14−18^ This technique enables the determination of the biomolecule concentration in a single droplet in buffer solutions and can be applied to quantify the local crowding environments in a living cell.

Applications of Raman measurements to liquid droplets in buffer solutions have been recently reported;^14,15,19−22^ however, their application to intracellular droplets is very limited. One drawback of Raman measurements is the low scattering cross section, which results in a low signal level. The presence of small Raman bands derived from various biomolecules in cells complicates the analysis and hinders small organelle imaging. Therefore, apart from nucleoli,^23^ the observation of intracellular liquid droplets with Raman imaging has been conducted only on fixed cells.^24^

In this study, we combined Raman and near-infrared (IR) fluorescence imaging to observe and quantify stress granules (SGs) formed in living cells under oxidative or hyperosmotic stress (Figure S1 in the Supporting Information). SGs are liquid droplets formed transiently as part of cellular responses to various stresses.^25,26^ SGs contain mRNA and various proteins, including ribonucleoproteins, and are regarded as membraneless organelles playing a crucial role in mRNA metabolism and translational control under stress.^27,28^ Moreover, SGs have been implicated in various diseases.^29,30^ Despite widespread research, quantitative analysis of intracellular SGs has not been explored due to the lack of noninvasive assays.

Distinguishing SGs from the surrounding cytoplasmic regions through refractive index is challenging,^31,32^ which hinders the identification of SGs when using bright field or phase contrast microscopy. To observe SGs using a Raman microscope, we used cells transfected with iRFP-G3BP1, in which G3BP1, one of the SG scaffold proteins, was labeled with the near-IR fluorescent protein iRFP713. With iRFP labeling, the location of SGs in living cells can be determined without interfering with Raman measurements with visible laser excitation (Figure S2). In this study, we acquired near-IR fluorescence images and determined the location of SGs within living cells, then performed Raman imaging of the SGs to perform *in situ* quantification of the components within the single SG and the surrounding cytoplasm. We compared the crowding environments within the SGs and the cytoplasm under different stresses, delving into the characteristics of intracellular droplets concerning crowding environments.

## Materials and Methods

### Cell Culture

HEK293A cells, expressing G3BP1 with iRFP fused to the C-terminus,^33^ were cultured in a glass-bottom dish (3960-035, 35 mm dish diameter, Matsunami), which was coated with 0.1 mg/mL poly-d-lysine (P7280, Merck), with 2 mL of culture medium (Dulbecco’s modified Eagle’s medium (DMEM) (D1145, Sigma) supplemented with 10% fetal bovine serum (10437-028, Gibco), 5 × 10^4^ U/L penicillin G, 50 mg/L streptomycin sulfate (15070-063, Gibco)), and 200 μg/mL hygromycin B (084-07681, Fuji Film). The cells were incubated overnight at 37 °C in a 5% CO_2_ humidified atmosphere. Before the Raman measurements, the culture medium was replaced with Hanks’ balanced salt solution (HBSS) (H2364, Sigma). To induce oxidative or hyperosmotic stress, cells were further incubated in HBSS containing 0.5 mM sodium arsenite (S080868, TRC)^34^ or 375 mM d-sorbitol (198-03755, Wako) for 30 min. The generation of SGs under the oxidative stress was confirmed using an immunofluorescence assay (Figure S3).

### Raman and Near-IR Fluorescence Imaging

Raman images were obtained with a confocal Raman system (Nanofinder flex2, Tokyo Instruments Inc.) combined with an inverted microscope (Eclipse Ti2, Nikon). The excitation light was 532 nm cw laser beam from a DPSS laser system (Sprout Solo, Lighthouse Photonics). A water-immersion objective lens (Plan Apo IR 60XC WI/NA = 1.27, Nikon) was used to excite the sample and collect Raman signals. The laser intensity at the entrance of the objective and the exposure time for each image point were 40 mW and 0.1 s, respectively. Before and after each Raman measurement, fluorescence images were obtained under the epi-illumination condition with a cooled sCMOS camera (CS-53M, Bitran) and an exposure time of 1.5 s. The excitation and observation wavelengths for the fluorescence measurements were 620 and 720 nm, respectively. All the measurements were performed at room temperature. The IGOR Pro program package (WaveMetrics) was used to analyze Raman and fluorescence images. The singular value decomposition analysis was adopted on each Raman image for noise reduction. Raman images were obtained by mapping the integrated intensity of the Raman band of interest.

### Raman Measurements of RNA Solution

Homogeneous yeast RNA solution (AM7118, Invitrogen) was purchased and used as a standard solution for making a calibration line. The solution was diluted with ultrapure water. The RNA concentration of the solution was determined by the absorbance at 260 nm. The solution was placed on a glass-bottom dish and the Raman spectra of the solution were measured using the Raman microscope.

## Results and Discussion

### Stress Granules under Oxidative Stress

**Figure 1:**
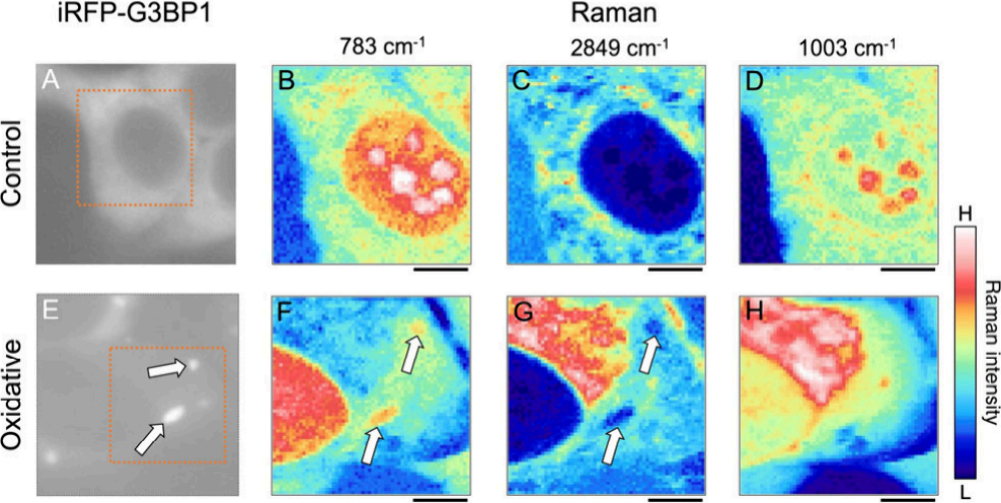
Near-IR fluorescence (A, E) and the corresponding Raman images of control (B–D) and oxidatively stressed (F–H) cells. Each Raman image was obtained by mapping the Raman intensity of the pyrimidine band (783 cm^–1^) (B, F), the CH_2_ symmetric stretching band (2849 cm^–1^) (C, G), and the phenylalanine band (1003 cm^–1^) (D, H). Orange boxes in the fluorescence images show the region of Raman imaging. White arrows in the images indicate stress granules. Scale bar: 5 μm.

Near-IR fluorescence and the corresponding Raman images of HEK293 cells with and without oxidative stress are shown in Figure 1 (another example is shown in Figure S4). In the control cells, the near-IR fluorescence exhibited a nearly uniform intensity throughout the cytoplasmic region, indicating that G3BP1 was widely distributed in the cytoplasm (Figure 1A). On the other hand, in the oxidative-stressed cells, bright spots were evident in the cytoplasm, attributed to SGs (Figure 1E and Figure S4A).

Raman images were generated by mapping the integrated intensities of Raman bands at 783 cm^–1^ (pyrimidine ring; nucleic acids), 1003 cm^–1^ (phenylalanine ring; proteins), and 2849 cm^–1^ (CH_2_ symmetric stretching (str.); lipids). These Raman bands were selected because they have low overlap with other bands and are isolated in Raman spectra of the cells. To generate Raman images, a straight line connecting two points around the peak of interest was subtracted from each spectrum as a baseline. It is worth noting that, in the nucleus, the amount of lipids is extremely small, whereas nucleic acids and proteins, which exhibit strong Raman bands at a higher wavenumber side (2900–3000 cm^–1^) of the C−H bands, exist with high concentration. Consequently, in the Raman images of lipids, the peak value becomes negative in the nucleus, which appears to contain less lipids than the outside medium. The Raman images of the stressed cell at 783 and 2849 cm^–1^ exhibited structures resembling the SGs in the near-IR fluorescence image (as denoted by arrows in Figure 1E, F, G and Figure S4A, B, C). Compared with the surrounding area, the Raman intensity was elevated at 783 cm^–1^ and reduced at 2849 cm^–1^. These images indicate that the nucleic acid concentration in the structured areas was higher than in the surrounding cytoplasmic region, whereas the lipid content was lower. These findings are consistent with the expected components of the SGs. Thus, successful Raman images of the SGs in living cells under oxidative stress were acquired by combining near-IR fluorescence with Raman measurements. In contrast, the Raman image at 1003 cm^–1^ did not reveal structures assignable to the SGs (Figure 1H and Figure S4D), suggesting that the protein concentration in the SGs was comparable to those in the surrounding area.

**Figure 2:**
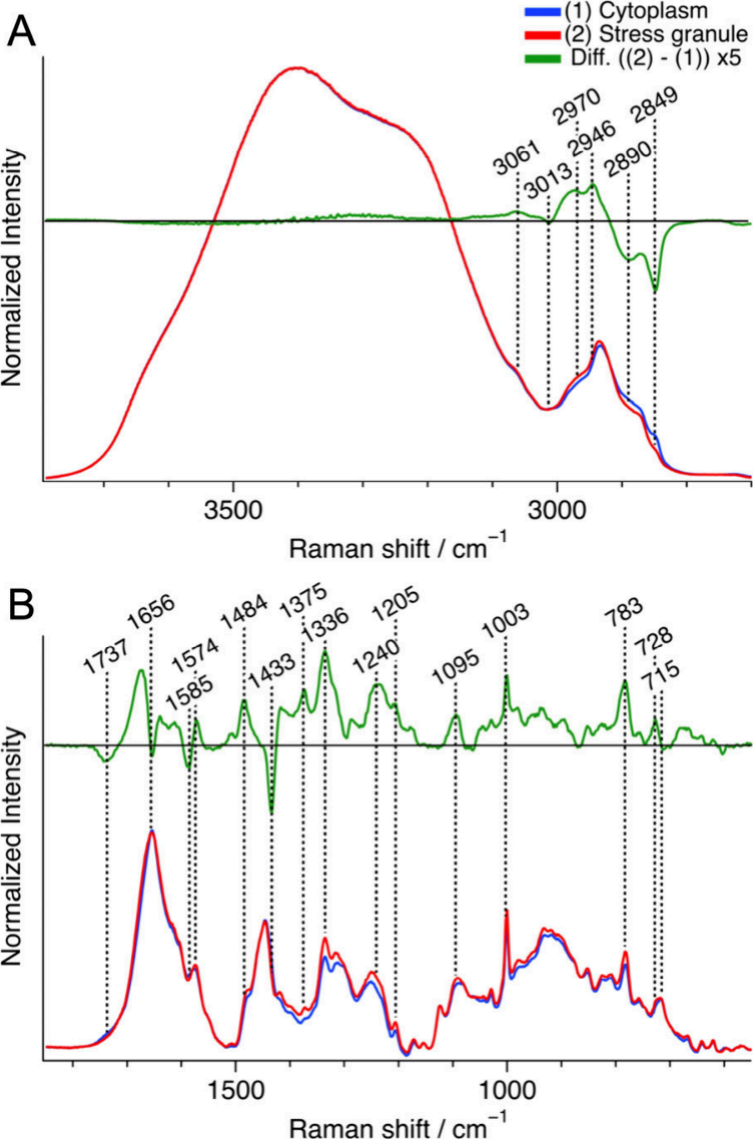
Representative average Raman spectra of the cytoplasm (blue, *n* = 7) and stress granules (red, *n* = 7) in oxidative stressed cells, along with their difference spectrum (granule – cytoplasm) (green) in the C–H and O–H stretching band region (A) and the fingerprint region (B). Each spectrum was normalized with the integrated intensity of the O–H stretching band (3200–3800 cm^–1^). For the fingerprint region (B), a baseline was obtained by fitting with a fourth-order polynomial and subtracted from each Raman spectrum, and the spectra were multiplied by 6 compared to those in (A). *n* represents the number of cells measured.

As shown in the Raman and fluorescence images, the cytoplasmic region is inhomogeneous even in control cells, possibly due to other organelles or concentration fluctuations. To compare the chemical components of SGs, the surrounding cytoplasm, and other regions, we calculated the average Raman spectrum of each region. We applied hierarchical clustering analysis to each Raman image, and, based on the results, the cell was divided into cytoplasm, nucleoplasm, nucleolus, and other regions (Figure S5). We then extracted the averaged Raman spectrum of each region from the Raman image. For SGs, on the other hand, the regions were chosen based on the corresponding fluorescence image, and the averaged Raman spectrum of the whole region of stress granules in each cell was obtained. Figure 2 shows the averaged Raman spectra of the SGs and the cytoplasm in the oxidative stressed cells, along with their difference spectrum (SGs – cytoplasm). The spatial resolution along the *Z*-axis was about 1 μm, and the spectra represent these spatial averages. The averaged Raman spectra were normalized with the integrated intensity of the water O–H band (3200–3800 cm^–1^) (Figure 2A), facilitating the evaluation of biomolecule concentrations relative to water concentration. The integrated below 3200 cm^–1^ was omitted to exclude the contributions of N–H and O–H bands of biomolecules. The difference spectrum in the fingerprint region (Figure 2B) showed negative peaks at 1737, 1656, 1433, and 715 cm^–1^, assigned to lipids, and positive peaks at 1484, 1336, and 783 cm^–1^, assigned to nucleic acids (Table S1). These negative and positive peaks confirmed that the interior of the SGs had a higher concentration of nucleic acids and a lower concentration of lipids compared to the cytoplasm under oxidative stress.

A positive peak was observed at 1003 cm^–1^ in the difference spectrum, indicating that the protein concentration in the SGs was higher than that in the surroundings. A pair of positive and negative peaks was found around the protein amide I band (1650–1700 cm^–1^). This differential-shaped peak can be attributed to the higher concentration of proteins and the lower concentration of lipids, which have a C=C str. band at 1656 cm^–1^, in the SGs. The positive peak in the amide I region also indicates that the protein concentration in the SGs was higher than in the surroundings. However, both the positive peak intensities at 1003 cm^–1^ and in the amide I region in the difference spectrum constituted approximately 10% of the corresponding intensities in the original spectra, indicating the difference in the total protein concentration was relatively small between the SGs and the cytoplasm. In the C–H str. region (2800–3000 cm^–1^), the intensity on the low and high wavenumber sides increased and decreased, respectively (Figure 2A). The C–H str. bands of lipids contributes to the low wavenumber side, and this result is consistent with the result in the fingerprint region.

**Figure 3:**
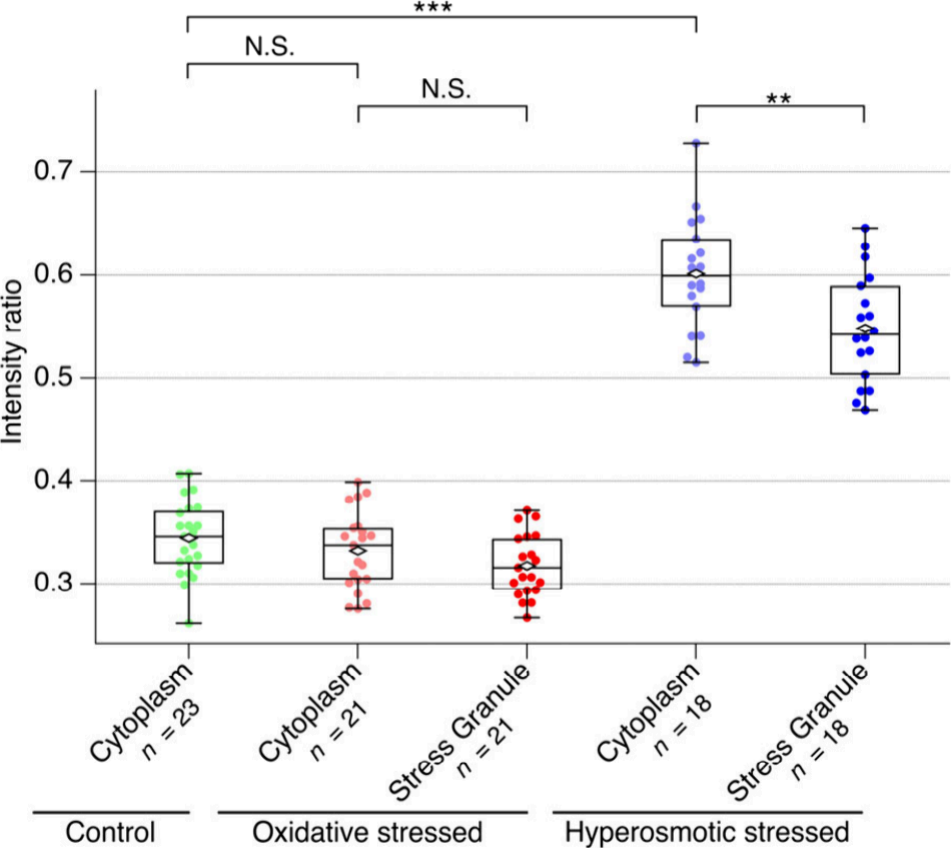
Intensity of the C–H stretching band (2800–3000 cm^–1^) relative to that of the O–H stretching band (3200–3800 cm^–1^) of the cytoplasm and the stress granules in control, oxidative, and hyperosmotic stressed cells. Open diamond and closed square marks are the averages and outliers, respectively. **: *p* < 0.01, ***: *p* < 0.001, N.S.: not significant. *n* represents the number of cells measured.

The intensity of the C–H str. bands relative to the water O–H band reflects the concentration of biomolecules in the cell. As shown in Figure 2A, the difference in the integrated intensity of the C–H str. bands between the SGs and the cytoplasm was minimal (<5%) (Figure 3). This means the total biomolecule concentration was nearly identical inside and outside the SGs; namely, the interior of the SGs formed via the oxidative stress was not highly condensed. The intensity of the O–H band of the intracellular region relative to that of the surrounding medium indicates no significant difference in water density between the SGs and the cytoplasm (Figure S6). These results indicate that the difference in the total biomolecular concentration was small between the SGs and the cytoplasm under the oxidative stress.

*In vitro* experiments on LLPS have shown that liquid droplets in a buffer solution are highly concentrated with specific biomolecules,^14,15^ which is apparently inconsistent with the present results showing a small difference in the biomolecular concentration between the SGs and the cytoplasm in living cells. The difference between *in vitro* and *in vivo* originates from the difference in the environment surrounding the droplets. While certain molecules, such as G3BP1, are concentrated in the SGs, similar to the liquid droplets in a buffer solution, at the same time, other molecules are excluded from the droplets; consequently, the net biomolecular concentration (i.e., the crowding environment) would be nearly identical inside and outside the SGs (Figure S7). LLPS in a living cell occurs as a rearrangement of biomolecular distribution inside a cell. Note that the present result is consistent with the result of refractive index measurements of SGs^31,32^ in which the refractive index inside the SGs was similar to that of the outside.

### Changes in Concentration of Biomolecules under Oxidative Stress

**Figure 4:**
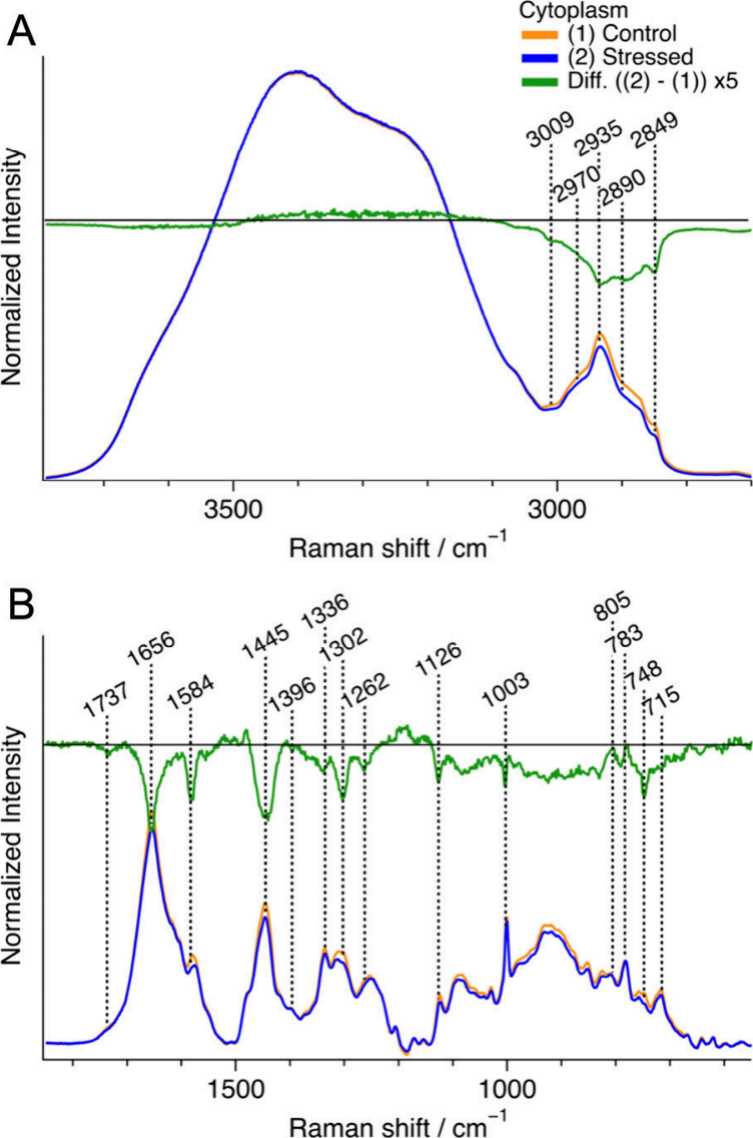
Representative average Raman spectra of cytoplasmic regions in control cells (yellow, *n* = 6) and oxidative stressed cells (blue, *n* = 7), along with their difference spectrum (oxidative – control) (green) in the C–H and O–H stretching band region (A) and the fingerprint region (B). Each spectrum was normalized with the integrated intensity of the O–H stretching band (3200–3800 cm^–1^). For the fingerprint region (B), a baseline was obtained by fitting with a fourth-order polynomial and subtracted from each Raman spectrum, and the spectra were multiplied by 6 compared to those in (A). *n* represents the number of cells measured.

We next compared the Raman spectra of the cytoplasm with and without the oxidative stress (Figure 4). A negative broad band was observed in the difference spectrum (oxidative – control) in the C–H str. band region (Figure 4A), indicating net biomolecule concentration decreased due to oxidative stress. The difference spectrum in the fingerprint region showed negative peaks attributed to lipids at 1737, 1656, 1445, and 715 cm^–1^, and to proteins at 1656, 1336, and 1003 cm^–1^, indicating both lipids and proteins decreased in the cytoplasm under the oxidative stress (Figure 4B). The magnitude of the decrease was less than 5%. The shape of the difference spectrum differs from the original, suggesting that the decrease in the concentration was not simply a result of the increase in the cell volume,^35^ rather, it can be attributed to cellular responses to the stress, such as lipid decomposition under stress and the degradation of oxidized proteins by autophagy.

Negative peaks were observed at 1584, 1126, and 748 cm^–1^, which are all assigned to the resonance Raman bands of the reduced form of cytochrome *c*, suggesting stress-induced mitochondrial dysfunction. Mitochondria play an important role in lipid homeostasis, and their dysfunction could also contribute to the decrease in lipid concentration. Changes in the intensity of the C–H str. band region in the nucleoplasm (Figure S8) and nucleoli (Figure S9) were less than a half of the change in the cytoplasm, implying the environment inside the nucleus was relatively stable against oxidative stress.

### Stress Granules under Hyperosmotic Stress

**Figure 5:**
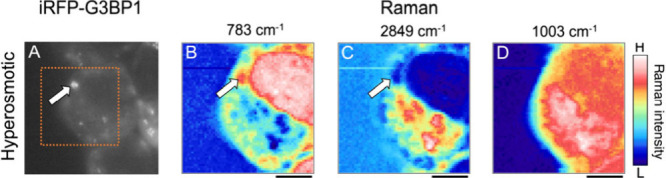
Near-IR fluorescence (A) and the corresponding Raman images of a hyperosmotic stressed cell (B–D). Each Raman image was obtained by mapping the Raman intensity of the pyrimidine band (783 cm^–1^) (B), the CH_2_ symmetric stretching band (2849 cm^–1^) (C), and the phenylalanine band (1003 cm^–1^) (D). Orange box in the fluorescence image shows the region of Raman imaging. White arrows in the images indicate stress granules. Scale bar: 5 μm.

As an alternative approach, we applied hyperosmotic stress to cells by adding sorbitol into the medium and conducted near-IR fluorescence and Raman imaging. As shown in Figure 5 (another example is shown in Figure S10), Raman images using the pyrimidine and lipid bands showed that in the hyperosmotic stress cells, similar to the oxidative stress cells, nucleic acids and lipid concentrations in the SGs were higher and lower, respectively, than those in the surrounding cytoplasm.

**Figure 6:**
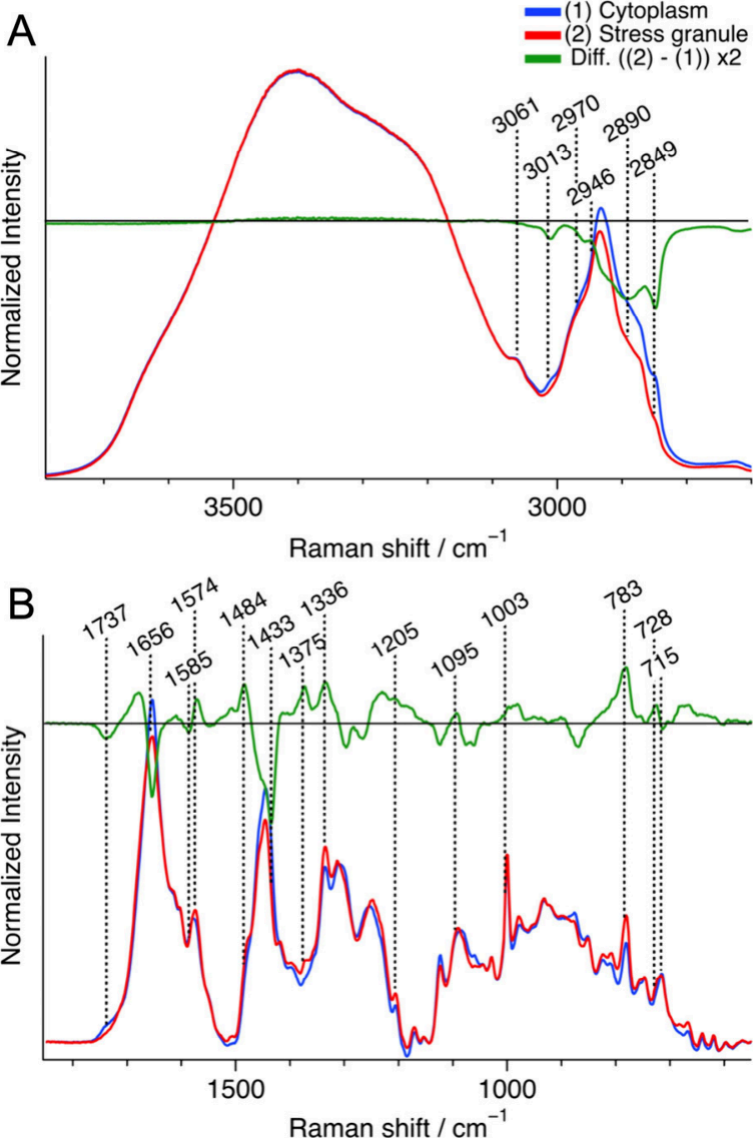
Representative average Raman spectra of the cytoplasm (blue, *n* = 6) and the stress granules (red, *n* = 6) in hyperosmotic stressed cells, and their difference spectrum (granule – cytoplasm) (green) in the C–H and O–H stretching band region (A) and the fingerprint region (B). Each spectrum was normalized with the integrated intensity of the O–H stretching band (3200–3800 cm^–1^). For the fingerprint region (B), a baseline was obtained by fitting with a fourth-order polynomial and subtracted from each Raman spectrum, and the spectra were multiplied by 6 compared to those in (A). *n* represents the number of cells measured.

However, the relationship between the Raman spectra of the SGs and the cytoplasm in the hyperosmotic stressed cells differed from that in the oxidative stressed cells (Figure 6). In hyperosmotic stressed cells, the intensity of the C–H str. bands in the SGs was lower than that in the surrounding cytoplasm. This result indicates that the total concentration of biomolecules inside the SGs was lower than in the surrounding areas. Under the hyperosmotic stress, the entire interior of cells becomes more crowded due to the stress-induced contraction,^11^ resulting in higher C–H band intensity compared to control cells (Figure 3). The cell contraction was also confirmed by a decrease in intracellular water density in the cytoplasm (Figure S6). The SGs under the hyperosmotic stress were also more crowded than those under the oxidative stress.

In cells subjected to hyperosmotic stress, the interior of the SGs was found to be sparser than the surroundings, forming a void-like structure. This result implies that, under contracted and highly dense conditions, the crowding environment in the SGs is rather maintained free from the hyperosmotic stress; the preservation of the crowding environment in the SGs may protect biomolecules inside the SGs against the cell contraction. These results indicate that the relationship between the degree of the crowding inside and outside the SGs depends on the type of stress.

In the difference spectrum (Figure 6), positive bands at 1484, 1375, 1336, 1095, and 783 cm^–1^ indicate that the nucleic acid concentration in the SGs was 50% higher than that in the cytoplasm. Negative bands at 1737, 1656, and 715 cm^–1^ exhibit a lower amount of lipids in the SGs, while the absence of clear bands at 1585 and 1003 cm^–1^ implies that the protein concentration inside the SGs was similar to the surroundings. The near IR fluorescence image shows that G3BP1 was markedly concentrated in the SGs (Figure 5A), indicating that other proteins were excluded from the SGs under hyperosmotic stress.

### Quantification of Concentration of Nucleic Acids

Finally, we conducted an *in situ* evaluation of the nucleic acid concentrations in the SGs and other regions in a cell. In our previous reports,^14,15^ the water Raman band was used as an intensity standard for determining protein concentration in a single liquid droplet in a buffer solution from the Raman spectra. In this study, we used the water Raman band of the medium surrounding cells as an intensity standard because the density of water in the buffer remained constant regardless of the cell sample.

The pyrimidine band at 783 cm^–1^ was used to obtain the concentration of nucleic acids. We first prepared a calibration line by measuring the Raman spectra of RNA solutions with varying concentrations (Figure S11). The intensity of the pyrimidine band relative to that of the O–H band (3200–3800 cm^–1^) was plotted against RNA concentration, providing a calibration line for the concentration quantification (Figure S12). Next, we calculated the intensity ratio of the pyrimidine band in each cellular compartment to the O–H band of extracellular water. We then converted these intensity ratios into the nucleic acid concentrations using the calibration line. In both the Raman spectra of aqueous solution and cells, a straight line was subtracted to remove effects of a broad baseline. Note that care should be taken when using the Raman band of intracellular water as an intensity standard because the water density in cells is not uniform and the intensity of the cellular O–H band differs from that of the medium (Figure S6). The Raman intensity of a solute is proportional to its concentration and in general independent of other solutes (Figure S13), so as long as the molecular structure remains the same, the calibration line using the Raman band of water as an intensity standard can be used to determine the nucleic acid concentration.

**Figure 7:**
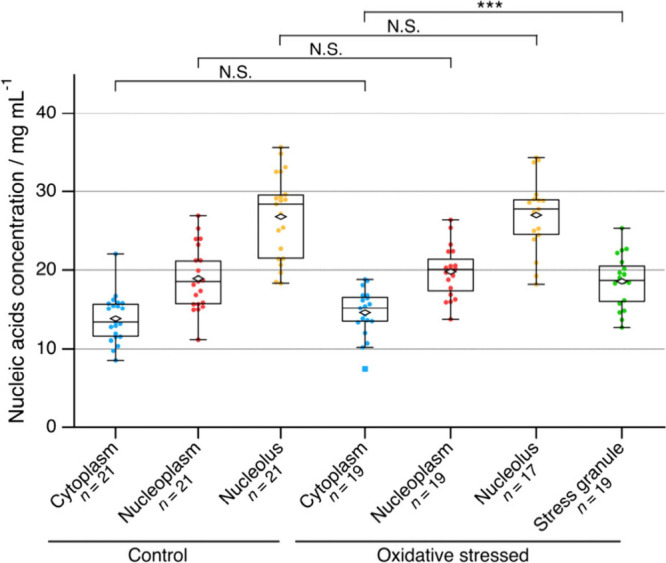
Nucleic acid concentrations in the cytoplasm, nucleoplasm, nucleoli, and stress granules in control and oxidative stressed cells. Diamond and square markers are averages and outliers, respectively. ***: *p* < 0.001, N.S.: not significant. *n* represents the number of cells measured.

Figure 7 shows the nucleic acid concentrations evaluated for different cellular regions in living cells with and without the oxidative stress. The concentrations of nucleic acids in the cytoplasm, nucleoplasm, and nucleoli in control living cells were evaluated to be around 14, 19, and 27 mg/mL, respectively. The volume of an animal cell is typically about 1 pL, indicating the total amount of nucleic acids in a cell is few tens picograms, which is comparable with the previous report of stained cells using deep-UV absorption microscopy.^36,37^ Changes in nucleic acid concentrations were minor following the oxidative stress in the cytoplasm, nucleoplasm, and nucleoli. The nucleic acid concentration in the SGs was 19 mg/mL, which is 20% higher than that in the surrounding cytoplasm and similar to that in the nucleoplasm. This result quantitatively shows that nucleic acids were localized to some extent in the SGs, while the nucleic acid concentration remained almost constant in other regions following the oxidative stress. Finally, we here obtained the spatial average of the concentrations of the organelles. When concentrations are estimated by other methods, such as mass spectrometry, the spatial average of each organelle is used and can be compared with this study.

### Liquid Droplets in Intracellular Molecular Crowding Environment

Nucleic acids were found to be concentrated within the SGs under both oxidative and hyperosmotic stress conditions. However, this does not imply that the total biomolecule concentration in the SGs is higher than that in the surrounding cytoplasm. This study indicates that the total biomolecular concentration in the SGs depends on the surrounding intracellular environment exhibiting molecular crowding conditions. Under oxidative stress, the total biomolecular concentration inside the SGs was similar to that of the surrounding cytoplasm, and the crowding environment inside and outside the SGs remained nearly identical. However, under hyperosmotic stress, while the cytoplasm became more crowded, the inside of the SGs was relatively maintained (Figure S7). These results indicate that a liquid droplet inside a cell does not necessarily represent a state of high biomolecular concentration. Under cellular stress, certain biomolecules assemble to form SGs, and, at the same time, some biomolecules are excluded from the SGs. The total biomolecular concentration within the SGs is similar to that of the surroundings under the oxidative stress and lower under the hyperosmotic stress. Our results exhibit the importance of considering a liquid droplet from the perspective that LLPS is a localization of specific biomolecules and the resulting droplet is not always in a dense state with a higher biomolecular concentration than its surrounding area.

RNA can play a key role in determining the properties of intracellular droplets.^38^ The total concentration of biomolecules, i.e., the degree of crowding environments, inside the SGs was reported to be regulated by RNAs.^32,39^ An excess volume of RNAs in the SGs is suggested to recruit RNA-binding proteins, including G3BP1, to form a mesh-like network inside the SGs, preventing the entry of large macromolecular complexes and thus maintaining the degree of crowding inside the SGs. In fact, the concentration of condensates of RNA and G3BP1 in a buffer solution is lower than those of protein condensates, suggesting the formation of mesh-like structures in RNA–G3BP1 complexes.^32,39^ This study shows that the nucleic acid concentration inside the SGs was higher than the surroundings under both stresses. Most of the observed nucleic acids are considered to be RNAs due to the RNA-binding property of G3BP1. This study provides the first label-free and direct confirmation of the presence of large amounts of RNAs in the SGs that regulate the internal crowding environment.

## Conclusion

We investigated liquid droplets formed by LLPS in a living cell, focusing on concentration and local crowding environment. The Raman spectra of the SGs quantitatively showed that the nucleic acid concentrations in the SGs were 20% and 50% higher than in the surrounding cytoplasm under the oxidative and hyperosmotic stresses, respectively. In contrast, the lipid concentration was lower than the surroundings. The net concentrations of biomolecules inside the SGs were altered depending on the type of stress. In the oxidative stressed cells, the total concentration inside the SGs was almost the same as in the cytoplasm, indicating that the crowding environment of the SGs was similar to the surrounding cytoplasm. In the hyperosmotic stressed cells, on the other hand, the interior of the SGs was less crowded than the exterior. These results indicate that SGs are not always dense granules formed by highly concentrated biomolecules, and their internal concentration varies depending on the surroundings and even becomes lower than the surroundings. The present study shows that intracellular LLPS can be regarded as a phenomenon in which biomolecules change their distributions in molecular crowding environments. Some molecules localize in a droplet, and some are excluded from the droplet, and the resulting droplets do not always have a higher total biomolecular concentration than their surroundings.

## Supporting Information Available

Schematic diagram of the experiment, Raman and iRFP fluorescence spectra, immunostained fluorescence images of stress granules, another examples of fluorescence and Raman images of cells under oxidative and hyperosmotic stresses, examples of HCA based cell classification, the density of water in cells, schematic diagram of the difference between *in vitro* and intracellular LLPS, Raman spectra of nucleoplasm and nucleolus in control and oxidative stressed cells, a Raman spectrum of aqueous RNA solution and a calibration line for RNA concentration, Raman spectra of RNA solution with and without protein, and assignments of Raman bands. The authors have cited additional references within the Supporting Information.^40−44^ (PDF)

## Accession Codes

The plasmid to express G3BP1-iRFP713 is deposited to RIKEN DNA bank (RDB17742) and Addgene (#129339).

## Abbreviations

LLPS: Liquid–liquid phase separation
SG: Stress granule
IR: Infrared
str.: stretching
