# Supplementary material for: Nucleic Acid-Rich Stress Granules Are Not Merely Crowded Condensates: A Quantitative Raman Imaging Study
Source: Anal Chem. 2024 Oct 15;96(43):17078–85. doi: 10.1021/acs.analchem.4c01096 (PMC11525929; doi:10.1021/acs.analchem.4c01096)
Supplement: Supplementary file 1 — ac4c01096_si_001.pdf [file ac4c01096_si_001.pdf]

Supporting Information

Nucleic Acid-Rich Stress Granules Are Not Merely Crowded Condensates: A Quantitative Raman Imaging Study

Ren Shibuya[§], Shinji Kajimoto[§,‡]*, Hideyuki Yaginuma[†,#], Tetsuro Ariyoshi[†,#], Yasushi Okada[†,#], and Takakazu Nakabayashi[§]*

[§]Graduate School of Pharmaceutical Sciences, Tohoku University, Aoba-ku, Sendai 980-8578, Japan.
E-mail: kajimoto@tohoku.ac.jp, takakazu.nakabayashi.e7@tohoku.ac.jp

[‡]JST PRESTO, Kawaguchi, Saitama 332-0012, Japan.

[†]Department of Cell Biology and Physics, Universal Biology Institute and International Research Center for Neurointelligence, The University of Tokyo, Bunkyo-ku, Tokyo 113-0033, Japan.

[#]Laboratory for Cell Polarity Regulation, Center for Biosystems Dynamics Research, RIKEN, Suita, Osaka 565-0874, Japan.

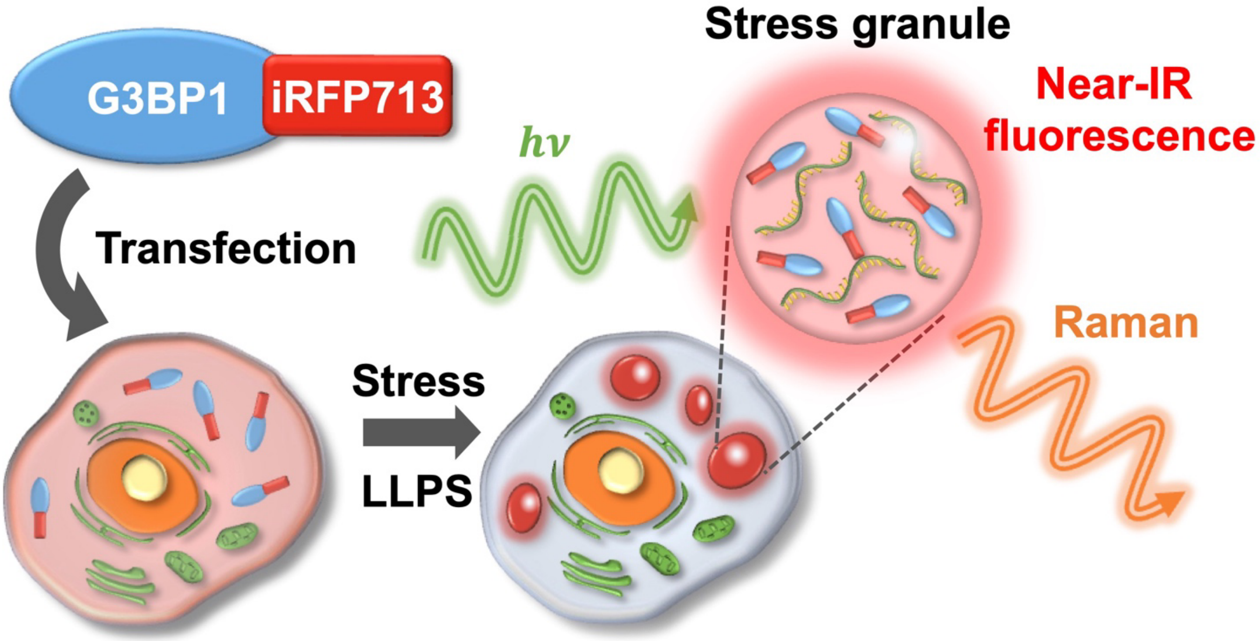

Figure S1. Schematic diagram of the experiments. iRFP713 fused G3BP1 protein was expressed in HEK293 cells. Then, cells were exposed to oxidative or hyperosmotic stress to induce stress granule formation. The positions of the stress granules were determined by near-IR fluorescence images, and Raman images of the stress granules were obtained.

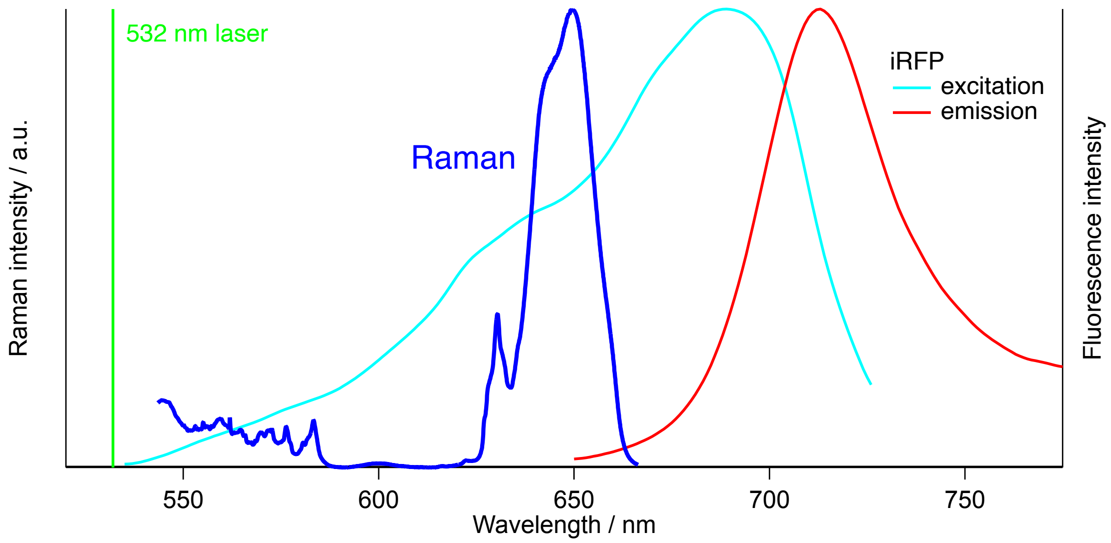

Figure S2. Spectral superimposition of Raman scattering obtained by 532 nm excitation light and iRFP fluorescence obtained by visible excitation light. Raman scattering is observed from 540 to 670 nm range. iRFP exhibits fluorescence around 720 nm[40, 41] , offering a clear window in the visible Raman region.

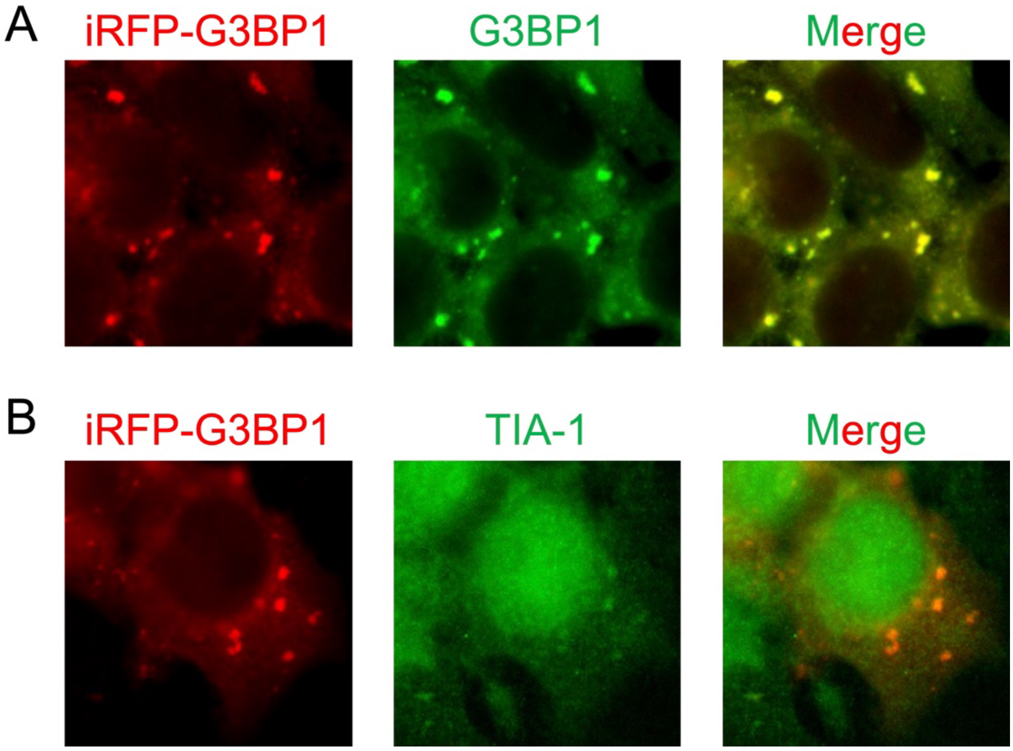

Figure S3. Near-IR fluorescence and immunostained fluorescence images of stress granules in iRFP-G3BP1 expressing HEK293A cells subjected to oxidative stress. Cells were treated with 0.5 mM sodium arsenite and then fixed and immunostained for G3BP1 (A) and TIA-1 (B) using G3BP1 (Invitrogen, PA5-29455) and TIA-1 (Invitrogen, PA5-76869) polyclonal antibodies, respectively, and donkey anti-rabbit IgG (H+L) highly cross-adsorbed secondary antibody, Alexa Fluor Plus 488 (Invitrogen, A32790). Note that Near-IR fluorescence and Raman images of HEK293 cells shown in the main text were obtained without fixation.

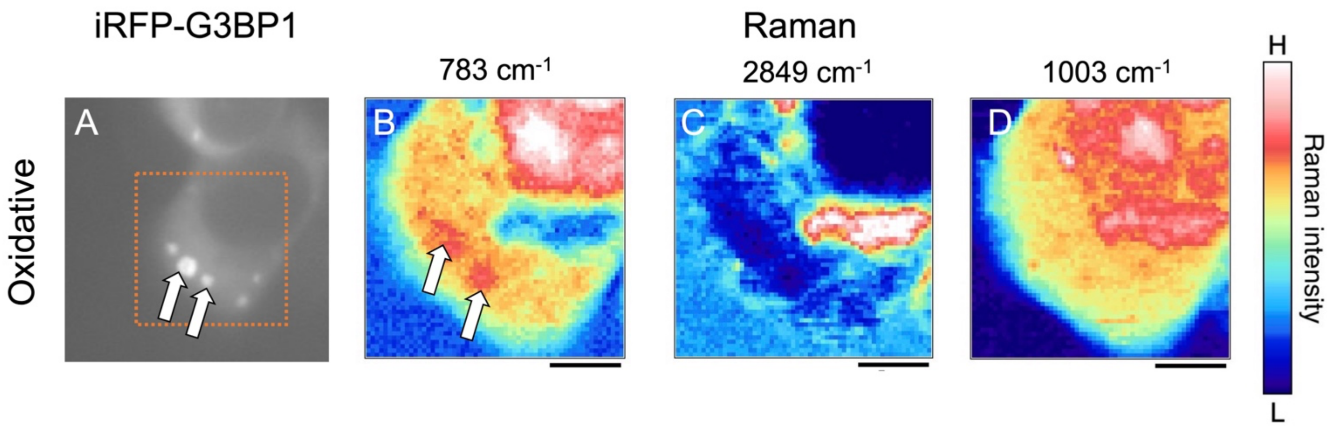

Figure S4. Another example of near-IR fluorescence (A) and the corresponding Raman images (B-D) of stress granules in an oxidative stressed cell. An orange box in the fluorescence image shows the region of the Raman imaging. White allows indicate the positions of stress granules. Each Raman image was obtained by mapping the Raman intensity of the pyrimidine band (783 cm^{-1}) (B), the CH_2 symmetric stretching band (2849 cm^{-1}) (C), and the phenylalanine band (1003 cm^{-1}) (D).

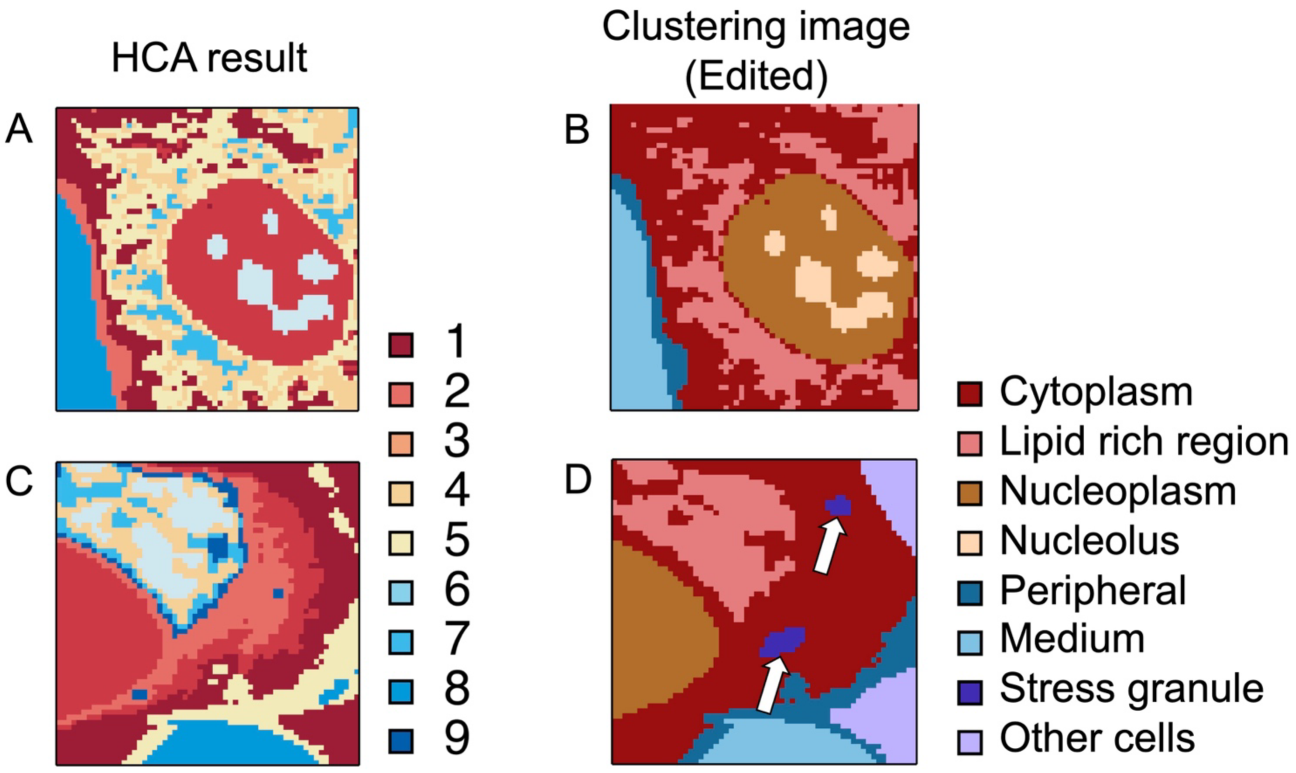

Figure S5. Examples of hierarchical cluster analysis (HCA) based cell classification. Based on the result of HCA of each Raman image (A, C), the cell was divided into cytoplasm, nucleoplasm, nucleolus, peripheral, and lipid and membrane-rich regions (B, D). For SGs, on the other hand, the regions were chosen based on the corresponding fluorescence image and the averaged Raman spectrum of whole region of stress granules in each cell was obtained. The corresponding fluorescence and Raman images were shown in Figure 1 in the main text.

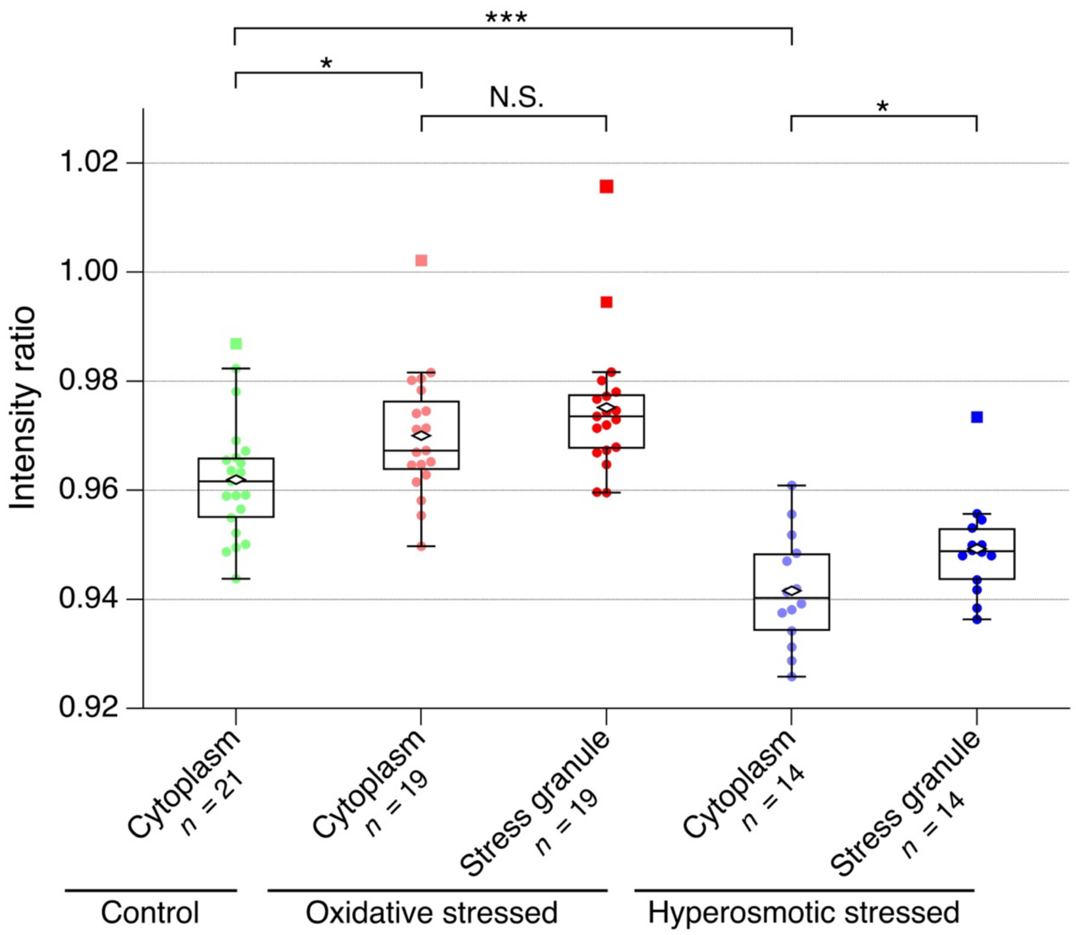

Figure S6. The density of water of control, oxidative, and hyperosmotic stressed cells. The water density of each compartment was estimated by the intensity ratio between the O–H stretching bands of the intracellular region and the surrounding medium. Diamond markers are averages and square markers are outliers. * : $p < 0.05$, ** : $p < 0.01$, N.S.: not significant. The smaller the intensity ratio, the lower the water density in the local cellular region, i.e., the higher the biomolecular concentration. Compared with control cells, oxidative stressed cells showed higher water density, indicating that the net concentration of biomolecules decreased upon the oxidative stress. There was no significant difference in the water densities in the SGs and cytoplasmic regions in the oxidative stressed cells. In hyperosmotic stressed cells, the intensity ratio of the cytoplasm was lower than that in the control cells, indicating that the interior of the cells became more crowded due to the contraction under the hyperosmotic stress. Under such condition, the intensity ratio inside the SGs was higher than the outside, indicating that the inside of the SGs rather maintained the crowding environment. *n* represents the number of cells measured.

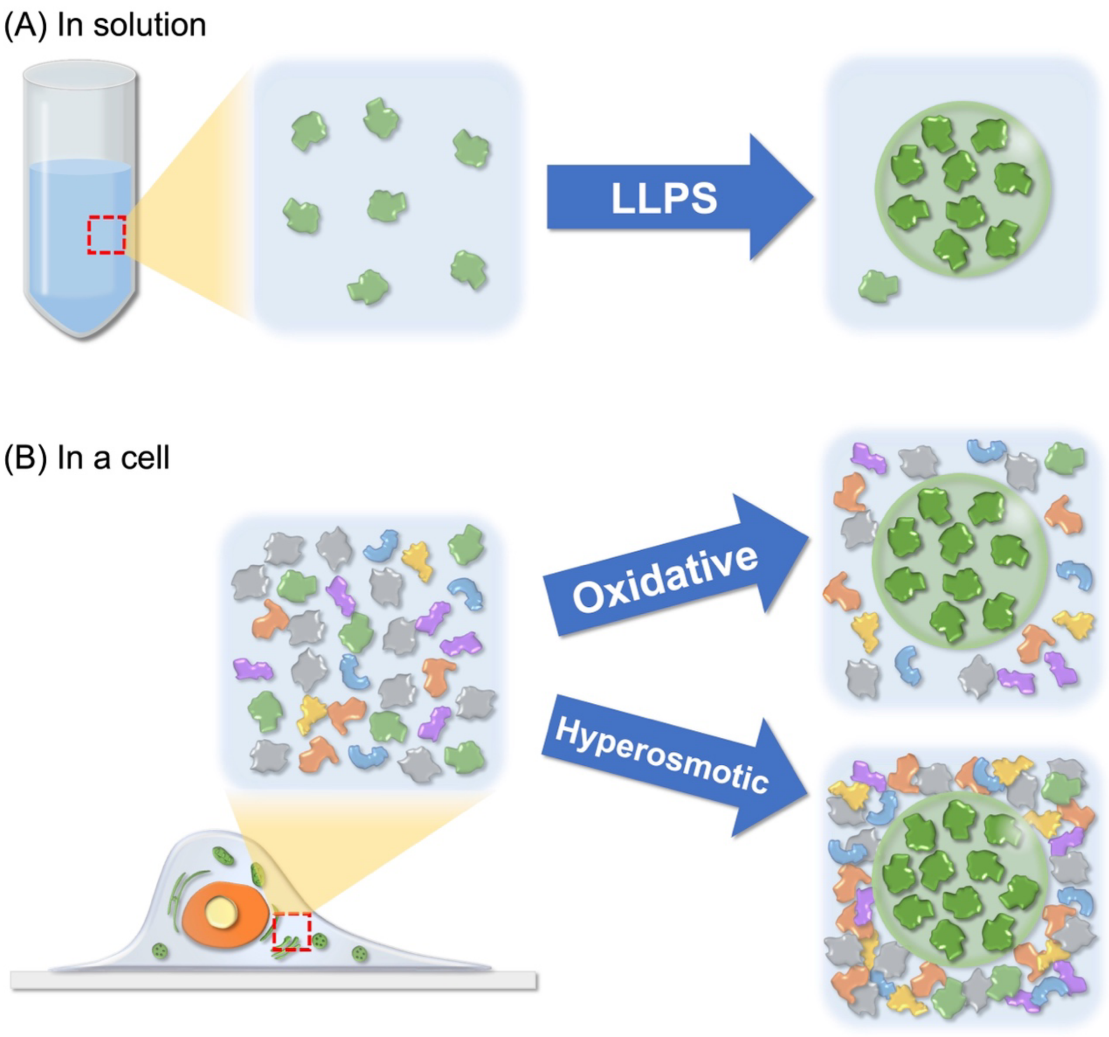

Figure S7. Schematic diagram of the difference between *in vitro* LLPS (A) and intracellular LLPS (B). While *in vitro* experiments provide a picture of highly concentrated droplets, the concentration of biomolecules in intracellular droplets is not necessarily high compared to the surrounding environment. In the case of stress granules formed by oxidative stress, only certain biomolecules relating to the droplet generation, such as G3BP1 and RNA, are concentrated, and the others are excluded, so the crowded environment inside the stress granules remains almost constant. In hyperosmotic stressed cells, the interior of entire cells becomes more crowded due to the contraction, but under such condition the inside of stress granules is rather maintained, forming “granules” that are sparser than the surrounding cytoplasm.

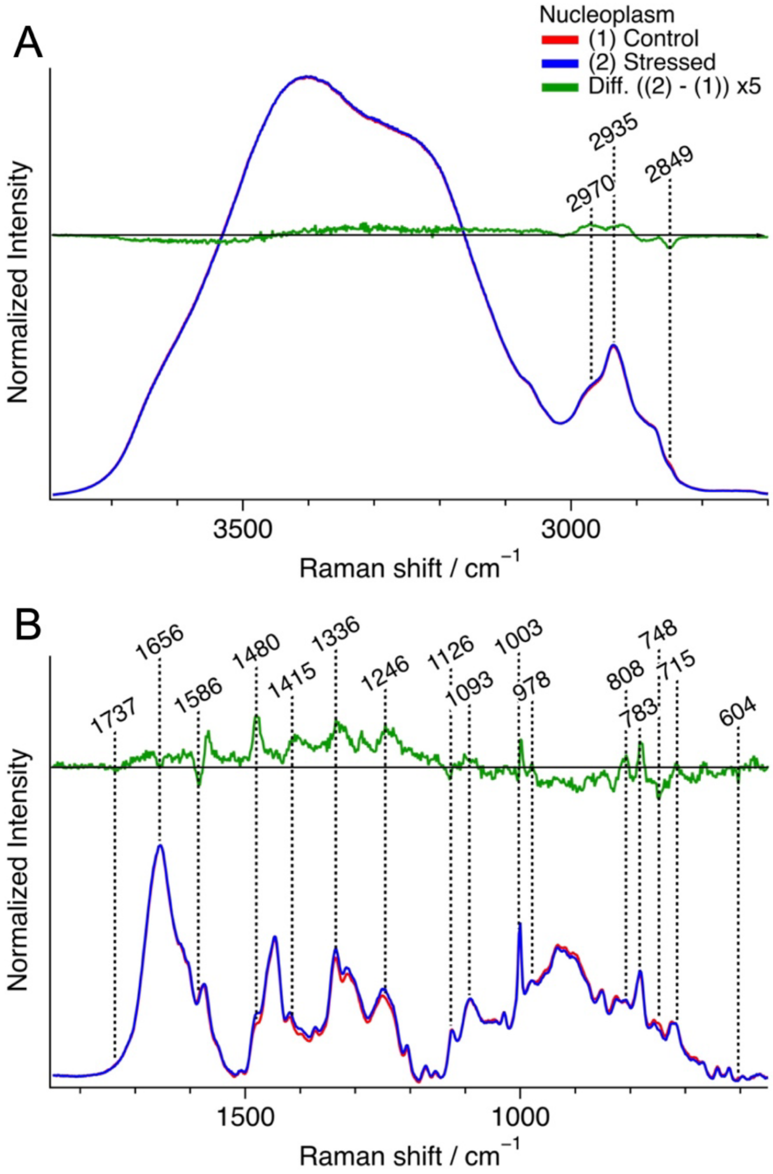

Figure S8. Representative average Raman spectra of nucleoplasm in control (red, n = 6) and oxidative stressed cells (blue, n = 7), and their difference spectrum (stressed – control) (green) in the C–H and O–H stretching band region (A) and the fingerprint region (B). Each spectrum was normalized with the integrated intensity of the O–H stretching band (3200–3800 cm^{-1}). For the fingerprint region, a baseline was obtained by fitting with a fourth-order polynomial and subtracted from each Raman spectrum, and the spectra were multiplied by 6 compared to those in the O–H and C–H stretching region. n represents the number of cells measured.

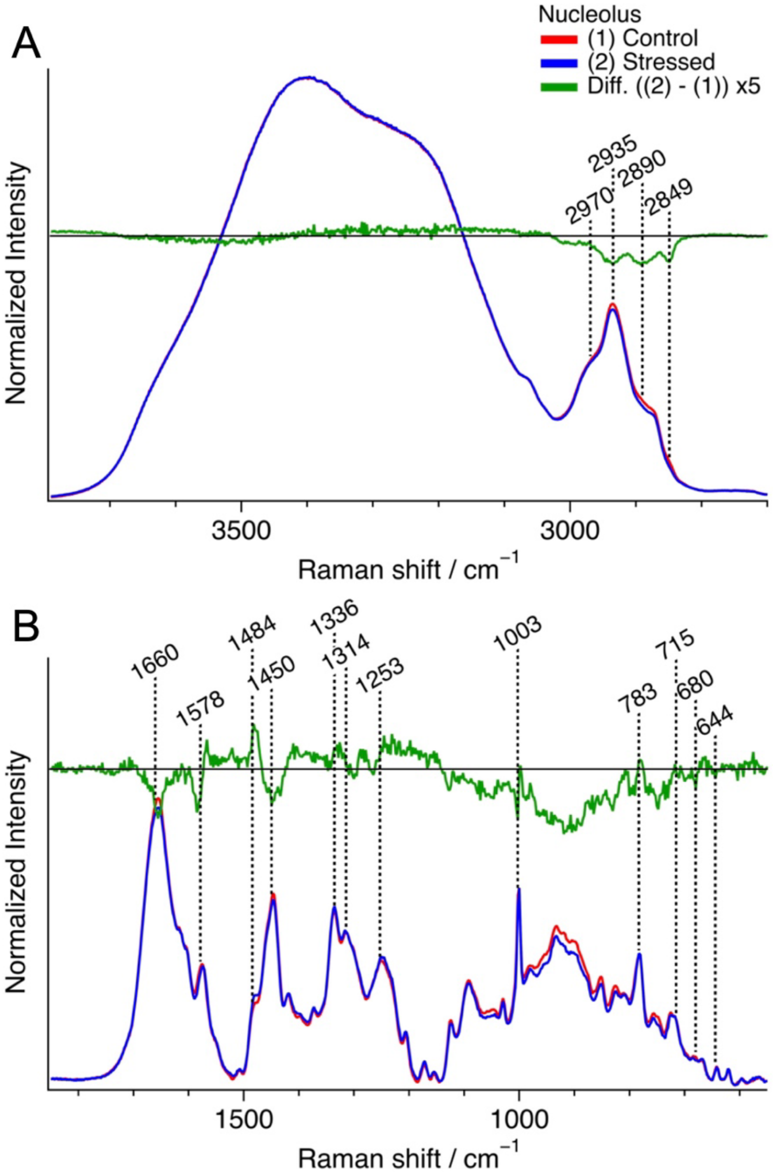

Figure S9. Representative average Raman spectra of nucleolus in control (red, n = 6) and oxidative stressed cells (blue, n = 5), and their difference spectrum (stressed – control) (green) in the C–H and O–H stretching band region (A) and the fingerprint region (B). Each spectrum was normalized with the integrated intensity of the O–H stretching band (3200–3800 cm^{-1}). For the fingerprint region, a baseline was obtained by fitting with a fourth-order polynomial and subtracted from each Raman spectrum, and the spectra were multiplied by 6 compared to those in the O–H and C–H stretching region. n represents the number of cells measured.

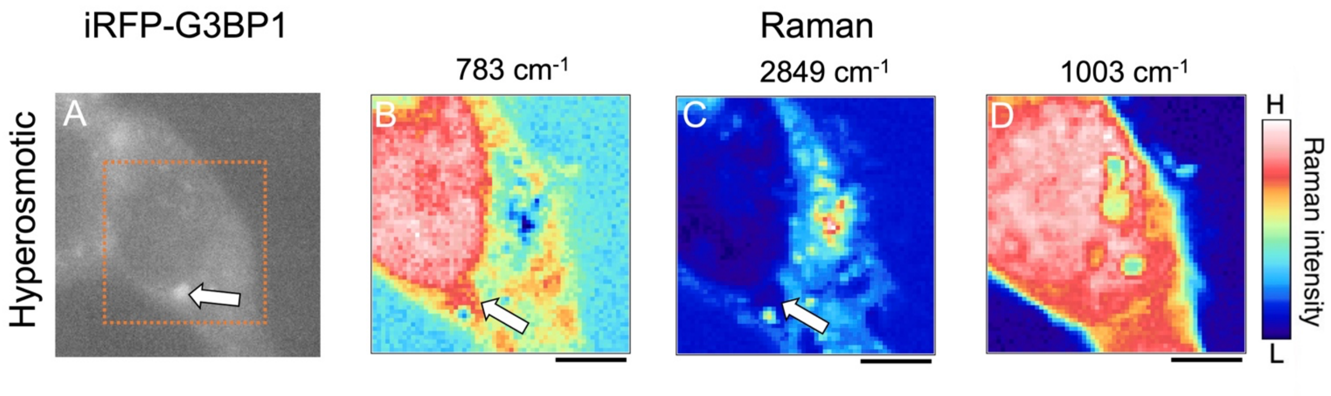

Figure S10. Another example of near-IR fluorescence (A) and the corresponding Raman images (B-D) of stress granules in a hyperosmotic stressed cell. An orange box in the fluorescence image shows the region of the Raman imaging. White allows indicate the positions of stress granules. Each Raman image was obtained by mapping the Raman intensity of the pyrimidine band (783 cm^{-1}) (B), the CH_2 symmetric stretching band (2849 cm^{-1}) (C), and the phenylalanine band (1003 cm^{-1}) (D).

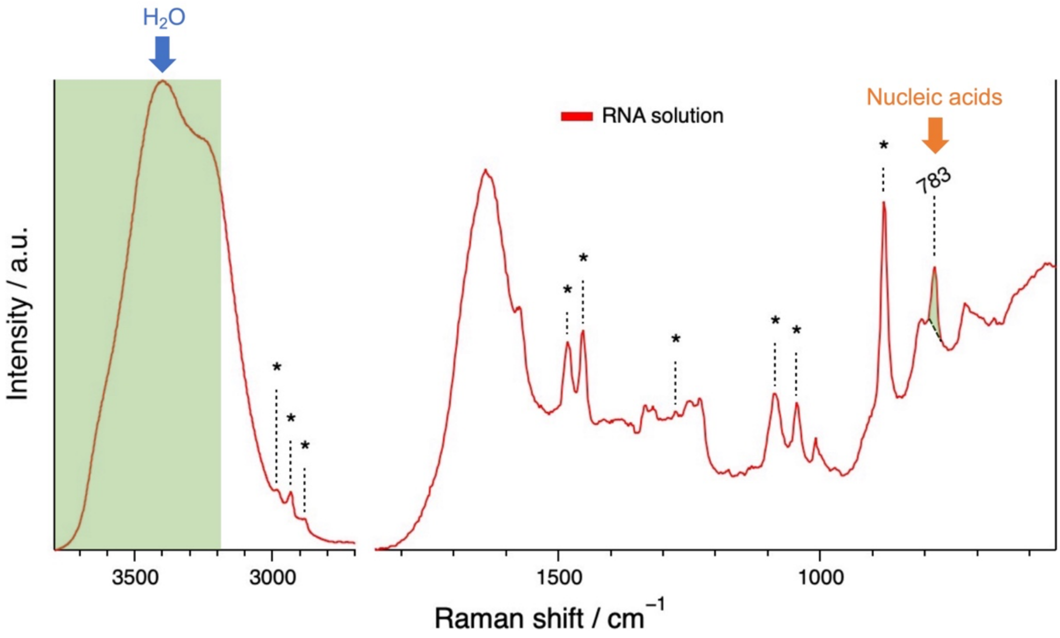

Figure S11. A typical Raman spectrum of an aqueous solution of RNA. Asterisks (*) show Raman bands assigned to ethanol.

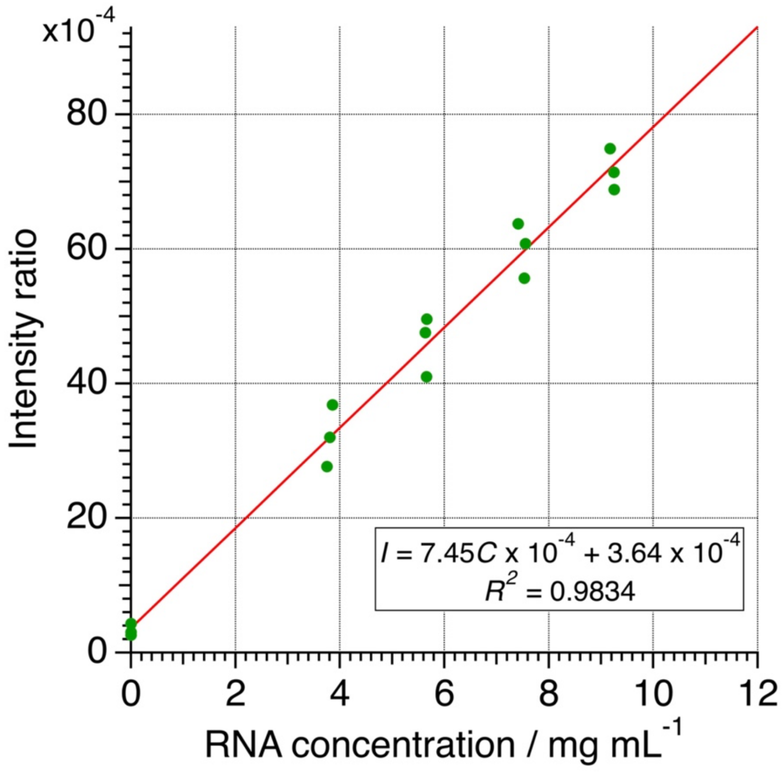

Figure S12. A calibration line for RNA concentration obtained by plotting the intensity ratio of the pyrimidine band at 783 cm^{-1} to the O–H stretching band of water at 3200–3800 cm^{-1} against RNA concentration.

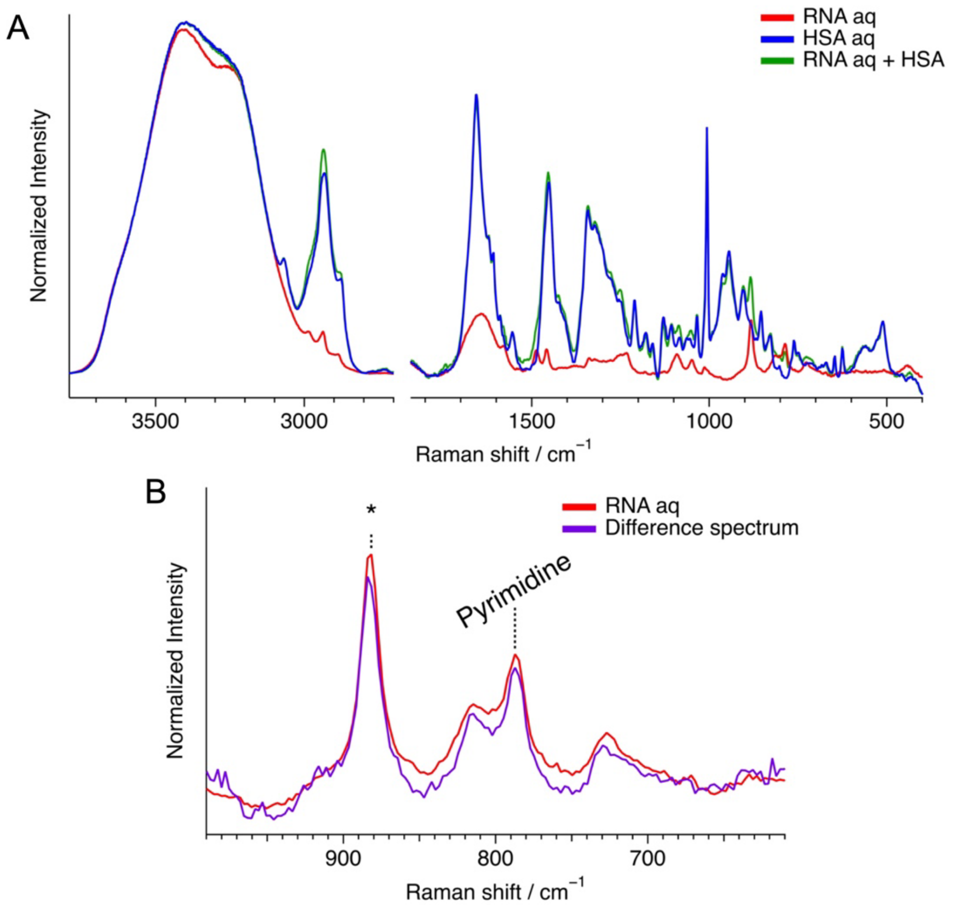

Figure S13. (A) Raman spectra of an RNA solution (10 mg/ mL, red), human serum albumin (HSA) solution (200 mg/mL, blue), and mixed solution containing both RNA and HSA (10 and 200 mg/ mL, respectively, green). (B) The difference spectrum (mixed solution – HSA solution, purple) superimposed with the Raman spectrum of the RNA solution (red) in the pyrimidine band region. The mixed solution was prepared by dissolving 20 mg of HSA in 100 μL of the 10 mg/mL RNA solution so that the concentration ratio of RNA to water remained the same after the HSA addition. All the spectra were normalized with the integrated intensity of the water O–H band (3450-3800 cm^{-1}). The fact that the peak height of the pyrimidine Raman band was the same between the Raman spectrum of the RNA solution and the difference spectrum indicates that the Raman intensity of RNA remains unchanged even in a solution containing 200 mg/mL of protein. The spectra in the fingerprint region in (A) was multiplied by 4 compared to the O–H stretching band region. An asterisk (*) in (B) shows the Raman band assigned to ethanol.

Table S1. Assignments of Raman bands[42-44].

A; adenine, T; thymine, G; guanine, C; cytosine, U; uracil, Phe; phenylalanine, Tyr; tyrosine, Trp; tryptophan, bk; backbone, def.; deformation, tw.; twist, sym.; symmetric, asym.; asymmetric, str.; stretch.

Raman shift / cm^{-1}	Assignments	Main molecular components
3061	aromatics	DNAs & RNAs, Proteins
3013 - 3009	=CH str.	Lipids
2970	CH_3 asym. str.	Proteins, Lipids
2946 - 2935	CH_3 sym. str.	Proteins, Lipids
2890	CH_2 asym. str.	Lipids
2849	CH_2 sym. str.	Lipids
1737	C=O	Lipids
1660 - 1653	C=C /Amide I	Lipids / Proteins
1586 - 1584		Cytochrome *c*
1578 - 1574	A, G	DNAs & RNAs
1484 - 1480	A, G	DNAs & RNAs
1450 - 1445	CH def.	Proteins, Lipids
1433	CH_2 def.	Lipids
1422 - 1415	A, G, bk	DNAs & RNAs
1396		Cytochrome *c*
1375	T	DNAs & RNAs
1336	A, G / CH def.	DNAs & RNAs / Proteins
1314	G / CH def.	DNAs & RNAs / Proteins / Cytochrome *c*
1302 - 1298	CH_2 tw.	Lipids
1267 - 1262	Amide III / =CH def.	Proteins / Lipids
1253	Amide III	Proteins
1246	Amide III	Proteins
1205	Phe, Trp, Tyr	Proteins
1126	C–N / C–C	Proteins / Lipids/ Cytochrome *c*
1095 - 1093	PO_2^-	DNAs & RNAs
1003	Phe	Proteins
978 - 976	C–C	Lipids
853	Tyr	Proteins
808 - 805	O–P–O	DNAs & RNAs / Lipids
790 - 783	U, T, C + bk (O–P–O)	DNAs & RNAs

748	Trp	Proteins / Cytochrome *c*
728	A	DNAs & RNAs
715	Choline	Lipids
680		Cytochrome *c*
644	Tyr	Proteins / cytochrome *c*
603		Cytochrome *c*
